# From mannan to bioethanol: cell surface co-display of β-mannanase and β-mannosidase on yeast *Saccharomyces cerevisiae*

**DOI:** 10.1186/s13068-016-0600-4

**Published:** 2016-09-02

**Authors:** Jun Ishii, Fumiyoshi Okazaki, Apridah Cameliawati Djohan, Kiyotaka Y. Hara, Nanami Asai-Nakashima, Hiroshi Teramura, Ade Andriani, Masahiro Tominaga, Satoshi Wakai, Prihardi Kahar, Yopi, Bambang Prasetya, Chiaki Ogino, Akihiko Kondo

**Affiliations:** 1 Graduate School of Science, Technology and Innovation, Kobe University, 1-1 Rokkodai, Nada, Kobe, 657-8501 Japan; 2 Organization of Advanced Science and Technology, Kobe University, 1-1 Rokkodai, Nada, Kobe, 657-8501 Japan; 3 Research Center for Biotechnology, Indonesian Institute of Sciences (LIPI), Cibinong Jalan Raya Bogor Km. 46, Cibinong, West Java 16911 Indonesia; 4 Department of Chemical Science and Engineering, Graduate School of Engineering, Kobe University, 1-1 Rokkodai, Nada, Kobe, 657-8501 Japan; 5 RIKEN Center for Sustainable Resource Science, 1-7-22, Suehiro, Tsurumi, Yokohama, 230-0045 Japan; 6 Department of Life Sciences, Graduate School of Bioresources, Mie University, 1577, Kurimamachiya, Tsu, Mie 514‑8507 Japan; 7 Department of Environmental Sciences, Graduate School of Nutritional and Environmental Sciences, University of Shizuoka, 52-1 Yada, Suruga, Shizuoka 422-8526 Japan

**Keywords:** Mannan, Yeast, *Saccharomyces cerevisiae*, Cell surface display, Mannanase, Mannosidase, Ethanol, Fermentation, Biomass resource, Biofuel

## Abstract

**Background:**

Mannans represent the largest hemicellulosic fraction in softwoods and also serve as carbohydrate stores in various plants. However, the utilization of mannans as sustainable resources has been less advanced in sustainable biofuel development. Based on a yeast cell surface-display technology that enables the immobilization of multiple enzymes on the yeast cell walls, we constructed a recombinant *Saccharomyces cerevisiae* strain that co-displays β-mannanase and β-mannosidase; this strain is expected to facilitate ethanol fermentation using mannan as a biomass source.

**Results:**

Parental yeast *S. cerevisiae* assimilated mannose and glucose as monomeric sugars, producing ethanol from mannose. We constructed yeast strains that express tethered β-mannanase and β-mannosidase; co-display of the two enzymes on the cell surface was confirmed by immunofluorescence staining and enzyme activity assays. The constructed yeast cells successfully hydrolyzed 1,4-β-d-mannan and produced ethanol by assimilating the resulting mannose without external addition of enzymes. Furthermore, the constructed strain produced ethanol from 1,4-β-d-mannan continually during the third batch of repeated fermentation. Additionally, the constructed strain produced ethanol from ivory nut mannan; ethanol yield was improved by NaOH pretreatment of the substrate.

**Conclusions:**

We successfully displayed β-mannanase and β-mannosidase on the yeast cell surface. Our results clearly demonstrate the utility of the strain co-displaying β-mannanase and β-mannosidase for ethanol fermentation from mannan biomass. Thus, co-tethering β-mannanase and β-mannosidase on the yeast cell surface provides a powerful platform technology for yeast fermentation toward the production of bioethanol and other biochemicals from lignocellulosic materials containing mannan components.

**Electronic supplementary material:**

The online version of this article (doi:10.1186/s13068-016-0600-4) contains supplementary material, which is available to authorized users.

## Background

Efficient utilization of renewable biomass resources is an important subject in the building of a sustainable society. Renewable plant constituents and residual biological wastes have great potential, since these materials constitute abundant and available resources [1, 2]. Various technologies permit the conversion of these sustainable resources into a wide range of compounds such as biofuels, biochemicals, and value-added products [2–5].

Bioethanol is a leading compound for sustainable biofuel development. As a first-generation biofuel, ethanol has been produced from starchy materials [6]. However, this route to bioethanol raises concerns regarding competition with food crops; therefore, research has focused on the conversion of lignocellulose to ethanol, a second-generation route to biofuel [6]. Lignocellulosic plants have a varying composition of macromolecules, with major components comprising abundance (on average) in the following order: glucan > lignin > xylan > mannan > arabinan > galactan [7]. Especially of interest for hydrolysis are the major sugar components glucan (from cellulose) and xylan (from hemicellulose) [2]; the resulting sugars are used as the substrates for subsequent fermentation [1, 2]. Arabinose and galactose also occasionally are utilized as the sugar substrates for fermentation [8, 9]. In contrast, mannose has to date rarely been utilized as the sugar substrate for fermentation.

Mannans are polysaccharides that consist of mannose as the major constituent unit. Mannans are classified into four subfamilies: linear mannan, glucomannan, galactomannan, and galactoglucomannan [10]. Each of these polysaccharides share in common a β-1,4-linked backbone containing mannose [10]. The mannans are widely distributed in nature as part of plant tissues [11, 12] and as constituents of glycoproteins in yeast cell walls [13]. In plants, mannans can be found as a component of hemicellulose and as a non-starch carbohydrate reserve [7]. As carbohydrate reserves, mannans are stored in endosperm walls and in vacuoles in seeds (e.g., coconut, coffee bean, locust bean) and in vacuoles in vegetative tissues (e.g., konjac, ivory nut, guar, aloe) [7, 10]. Representatives of these mannans include those found in aloe vera and ivory nut, in which the molecules are linear mannans consisting of linear main chains of 1,4-linked β-d-mannopyranosyl residues and contain less than 5 % galactose [10]. While these plants are widely used in the food industry as sources of mannan extracts, some other mannan-rich materials (such as coffee bean, palm kernel, and copra meal) are discarded as wastes in the food supply chain [7]. Thus, the mannans are of great interest as potential sustainable resources for biorefining.

Two major mannan-degrading enzymes are β-mannanase (1,4-β-d-mannan mannohydrolase, EC 3.2.1.78) and β-mannosidase (1,4-β-d-mannopyranoside hydrolase, EC 3.2.1.25) [7, 10, 14–18]. β-Mannanase is an endo-acting enzyme that catalyzes the random hydrolysis of β-1,4-linked internal linkages of the mannan backbone [10, 14–16]. β-Mannosidase is an exo-acting enzyme that cleaves β-1,4-linked mannosides from the non-reducing end of the chain [10, 17, 18]. These two enzymes act synergistically on mannans and manno-oligosaccharides to release mannose. Some microbes utilize the released monosaccharide, mannose, as a sugar substrate for subsequent fermentation [19].

Budding yeast (*Saccharomyces cerevisiae*) is a microorganism that has been used traditionally for ethanol fermentation. Recent advances in bioethanol production using yeast have employed simultaneous saccharification and fermentation (SSF) of the substrate, a process that severely impacts the cost effectiveness of biorefining from lignocellulosic materials [1, 2]. Notably, recent advances in yeast cell surface-display technology that permit the immobilization of multiple enzymes (such as cellulases) on the yeast cell wall have been successfully incorporated into the SSF process [2, 20–23]. However, this technology has been utilized predominantly to display cellulases and xylanases on yeast cell surfaces, and have not been applied to tether the mannan-degrading enzymes for use in the SSF processes.

**Fig. 1:**
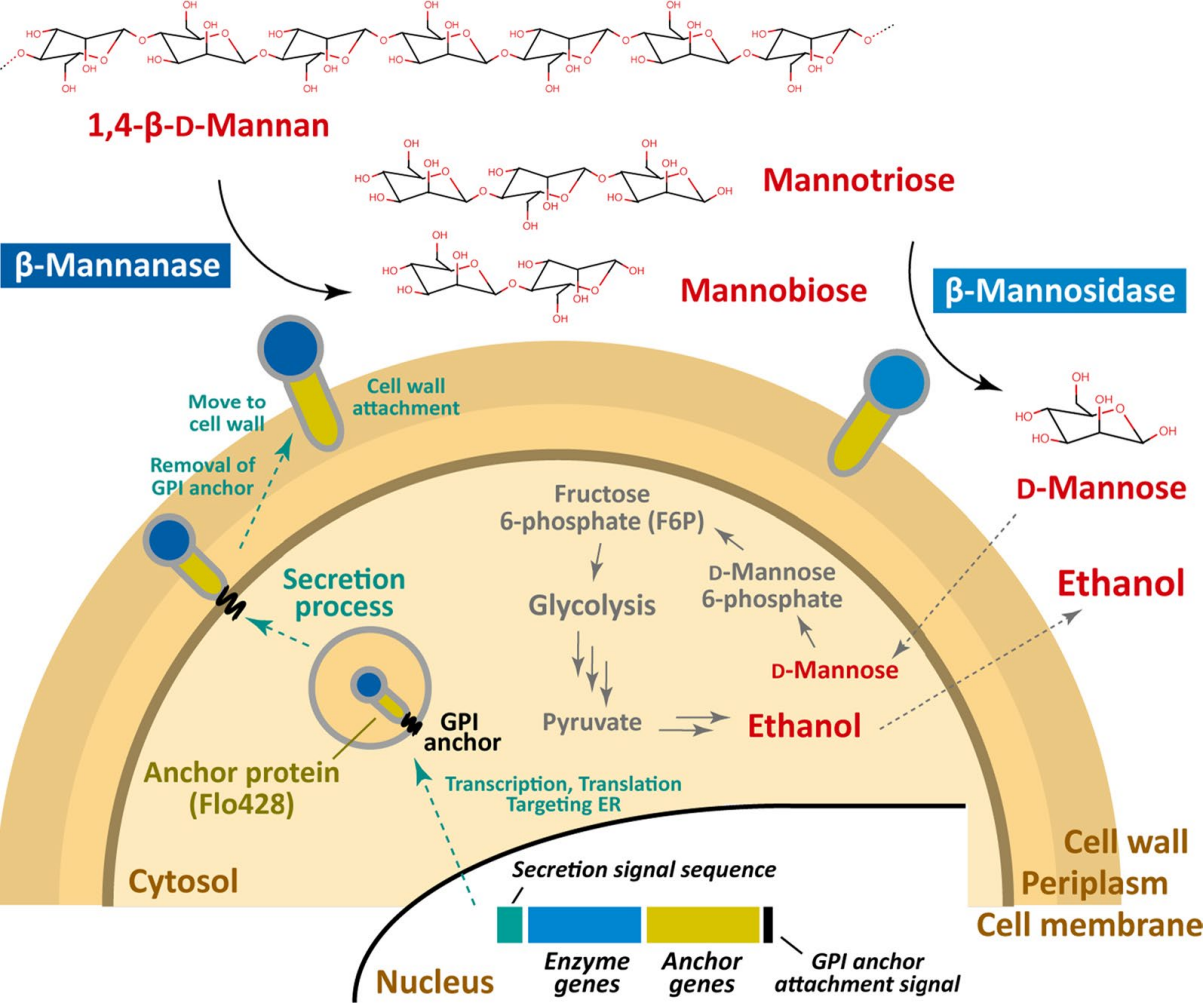
Schematic overview of β-mannanase and β-mannosidase display on the cell wall of *S. cerevisiae* for ethanol production from 1,4-β-d-mannan. For the yeast cell surface display of the protein of interest, the genes encoding a secretion signal sequence and an anchor protein (containing GPI anchor attachment signal sequence) are fused to the target gene so as to encode fusions at the N-terminus and C-terminus, respectively. In the present study, the β-mannanase and β-mannosidase fused with the Flo428 anchor protein are used for display on the yeast cell surface. After processing via the secretion pathway, the fusion proteins migrate to the cell surface and are tethered on the yeast cell wall following the removal of the GPI anchor at the cell membrane. The displayed β-mannanase and β-mannosidase enzymes can degrade 1,4-β-d-mannan, and the host then can assimilate the resulting mannose for fermentation to ethanol

In the present study, we constructed a *S. cerevisiae* recombinant strain co-displaying β-mannanase and β-mannosidase; this yeast strain was expected to permit ethanol fermentation using mannan as a biomass resource (Fig. 1). We demonstrate that the engineered yeast cells successfully hydrolyze the linear mannans and produce ethanol by assimilation of mannose generated by enzymatic degradation of 1,4-β-d-mannan or of ivory nut mannan.

## Results and discussion

### Cell growth of *S. cerevisiae* using mannose as a carbon source

To test whether yeast is able to assimilate mannose as a sole carbon source, cell growth of *S. cerevisiae* YPH499 was investigated (Additional file 1: Figure S1). YPH499 cells were pre-cultured in synthetic dextrose (SD) minimal medium containing 2 % glucose. After collecting and washing, the cells were separately and inoculated into fresh SD minimal glucose medium and synthetic mannose (SM) minimal medium (containing 2 % mannose) to monitor cell growth. YPH499 yeast cells exhibited similar growth kinetics in SD and SM media, although the growth in SM medium was delayed compared to that in SD medium (Additional file 1: Figure S1). The delay in SM medium might be attributed to the change of carbon sources from glucose to mannose. The specific growth rates (μ) of YPH499 in early logarithmic phase were 0.327 ± 0.006 h^−1^ in SD medium and 0.303 ± 0.014 h^−1^ in SM medium (6–8 h), respectively (mean ± standard deviation).

### Construction of β-mannanase- and β-mannosidase-co-displaying yeast

In the most widely used yeast cell surface-display systems, target proteins are encoded with N-terminal secretion signal sequences and C-terminal anchor protein signal sequences [i.e., cell wall proteins containing glycosylphosphatidylinositol (GPI) anchor attachment sequences] (Fig. 1) [20, 24]. One such cell surface-display system using Flo1p (a lectin-like cell wall protein) permits the incorporation of an anchor ranging in length from 42 a.a. (Flo42) to 1326 a.a. (Flo1326), thereby accommodating accessibility of large substrates to the target enzymes displayed on the yeast cell wall [25]. Among the anchors, Flo428 (428 a.a.) is a well-balanced anchor protein that provides both substrate accessibility and enzyme expression [25–27].

To make the mannan-fermenting yeast strains, we chose the β-mannanase (Man5A) [14, 15] and β-mannosidase (Mnd2A) [18] from *Aspergillus aculeatus* as the mannan-degrading enzymes. The genes encoding Man5A and Mnd2A were amplified as open reading frames (ORFs) lacking the start codons and the stop codons; the *man5A* and *mnd2A* ORFs were amplified with downstream sequences encoding epitope tags (i.e., to encode C-terminal FLAG tag or HA tag, respectively). The resulting sequences were cloned into the multiple cloning sites of pFGK426 and pFGK424 yeast cell surface-display 2μ multi-copy vectors, respectively (Table 1). The resulting plasmids (pFGK426-AaMan5A and pFGK424-AaMnd2A) were designed to display Man5A and Mnd2A on the yeast cell surface using a prepro-α-factor secretion signal and a Flo428 anchor protein (Table 1). In parallel constructions, *man5A* and *mnd2A* ORFs were amplified without start codons but with stop codons following the respective epitope tag-encoding sequences. The ORFs were cloned into pFGK426 and pFGK424 (respectively), yielding plasmids (pFGK426-AaMan5A-TAA and pFGK424-AaMnd2A-TAA) that directed the extracellular secretion (without cell wall anchoring) of Man5A and Mnd2A (Table 1). Individual and paired plasmids, as well as the respective empty control (mock) plasmids (pFGK426 and/or pFGK424), were transformed into YPH499 (Table 2).

**Table 1:** Plasmids used in this study

Plasmid	Specific features	Source
pGK426	Yeast multi-copy expression vector containing *PGK1* promoter, *PGK1* terminator, 2*μ* origin, and *URA3* marker	[28]
pFGK426	Yeast cell surface-display vector using Flo428 anchor protein; contains coding sequences for secretion signal sequence of α-factor and Flo428 anchor (C-terminal 428 a.a. of Flo1p) in pGK426	[26]
pFGK426-AaMan5A	*A. aculeatus* β-mannanase (Man5A) display in pFGK426; fused with FLAG tag at the C-terminus	This study
pFGK426-AaMan5A-TAA	*A. aculeatus* β-mannanase (Man5A) secretion in pFGK426; fused with FLAG tag at the C-terminus	This study
pGK424	Yeast multi-copy expression vector containing *PGK1* promoter, *PGK1* terminator, 2*μ* origin, and *TRP1* marker	[28]
pFGK424	Yeast cell surface-display vector using Flo428 anchor protein; contains coding sequences for secretion signal sequence of α-factor and Flo428 anchor in pGK424	This study
pFGK424-AaMnd2A	*A. aculeatus* β-mannosidase (Mnd2A) display in pFGK424; fused with HA tag at the C-terminus	This study
pFGK424-AaMnd2A-TAA	*A. aculeatus* β-mannosidase (Mnd2A) secretion in pFGK424; fused with HA tag at the C-terminus	This study
pRS406	Yeast integration vector containing *URA3* marker	ATCC
pFGK406-AaMan5A	Yeast integration plasmid for displaying *A. aculeatus* β-mannanase (Man5A) using pFGK426 expression cassette	This study
pRS402	Yeast integration vector containing *ADE2* marker	ATCC
pFGK402-AaMnd2A	Yeast integration plasmid for displaying *A. aculeatus* β-mannosidase (Mnd2A) using pFGK424 expression cassette	This study

**Table 2:** Transformants used in this study

Strain name	Specific features
Mock/Mock	YPH499/pFGK426/pFGK424
Man5A-Disp/Mock	YPH499/pFGK426-AaMan5A/pFGK424
Mock/Mnd2A-Disp	YPH499/pFGK426/pFGK424-AaMnd2A
Man5A-Disp/Mnd2A-Disp	YPH499/pFGK426-AaMan5A/pFGK424-AaMnd2A
Man5A-Sec/Mock	YPH499/pFGK426-AaMan5A-TAA/pFGK424
Mock/Mnd2A-Sec	YPH499/pFGK426/pFGK424-AaMnd2A-TAA
Man5A-Sec/Mnd2A-Sec	YPH499/pFGK426-AaMan5A-TAA/pFGK424-AaMnd2A-TAA
Man5A-Disp-G/Mnd2A-Disp-G	YPH499/pFGK406-AaMan5A/pFGK402-AaMnd2A

**Fig. 2:**
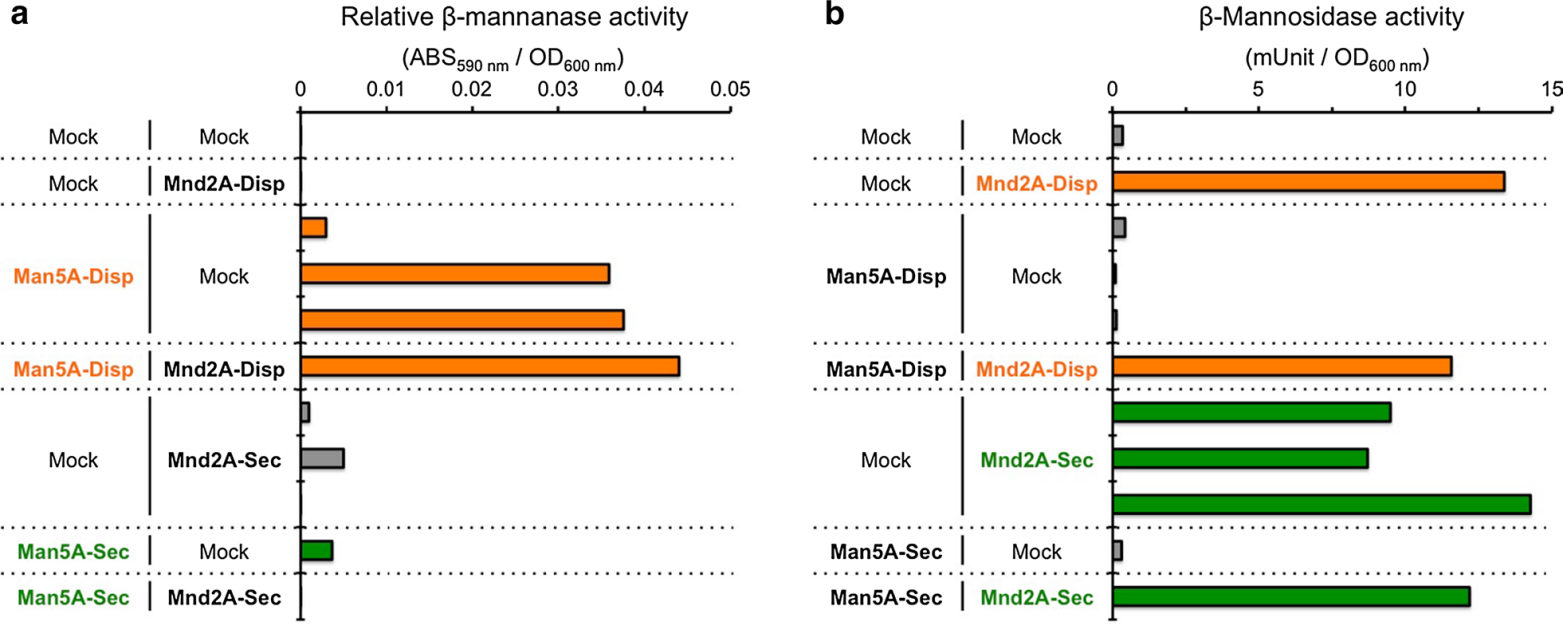
Enzyme activities of Man5A- and/or Mnd2A-displaying and -secreting yeast cells. **a** Relative β-mannanase activities of yeast transformants were measured using azo-carob galactomannan. *Orange* Man5A-displaying yeast cells. *Green* Man5A-secreting yeast cells. *Gray* Man5A-non-expressing yeast cells. **b** β-Mannosidase activities of yeast transformants were measured using *p*-nitrophenyl-β-d-mannopyranoside. One unit of β-mannosidase activity was defined as the amount of enzyme that liberated 1 μmol of *p*-nitrophenol per min under the above conditions. *Orange* the Mnd2A-displaying yeast cells. *Green* the Mnd2A-secreting yeast cells. *Gray* the Mnd2A-non-expressing yeast cells. Relative β-mannanase or β-mannosidase activities were calculated by normalizing the ABS_590_ values or the units, respectively, per OD_600_ unit

After cultivating the transformants in synthetic dextrose–casamino acids (SDC) selection media, the relative β-mannanase activities and the β-mannosidase activities of the yeast cells were measured using azo-galactomannan and *p*-nitrophenyl-β-d-mannopyranoside (*p*NP-mannopyranoside) as the respective substrates (Fig. 2). Two of three different colonies of Man5A-displaying yeast cells and Man5A/Mnd2A-displaying yeast cells showed β-mannanase activities, whereas other transformants (including Man5A- and Man5A/Mnd2A-secreting yeast cells) did not exhibit β-mannanase activities (Fig. 2a). In contrast, all Mnd2A-displaying and -secreting yeast cells exhibited similar β-mannosidase activities (Fig. 2b). This result might be attributed to the reaction of Mnd2A proteins on the cell surfaces during the secretion process or inside the cells through the uptake of the small substrate *p*NP-mannopyranoside. Nonetheless, our results confirmed that Mnd2A- and Man5A/Mnd2A-displaying yeast cells exhibited β-mannosidase activity.

**Fig. 3:**
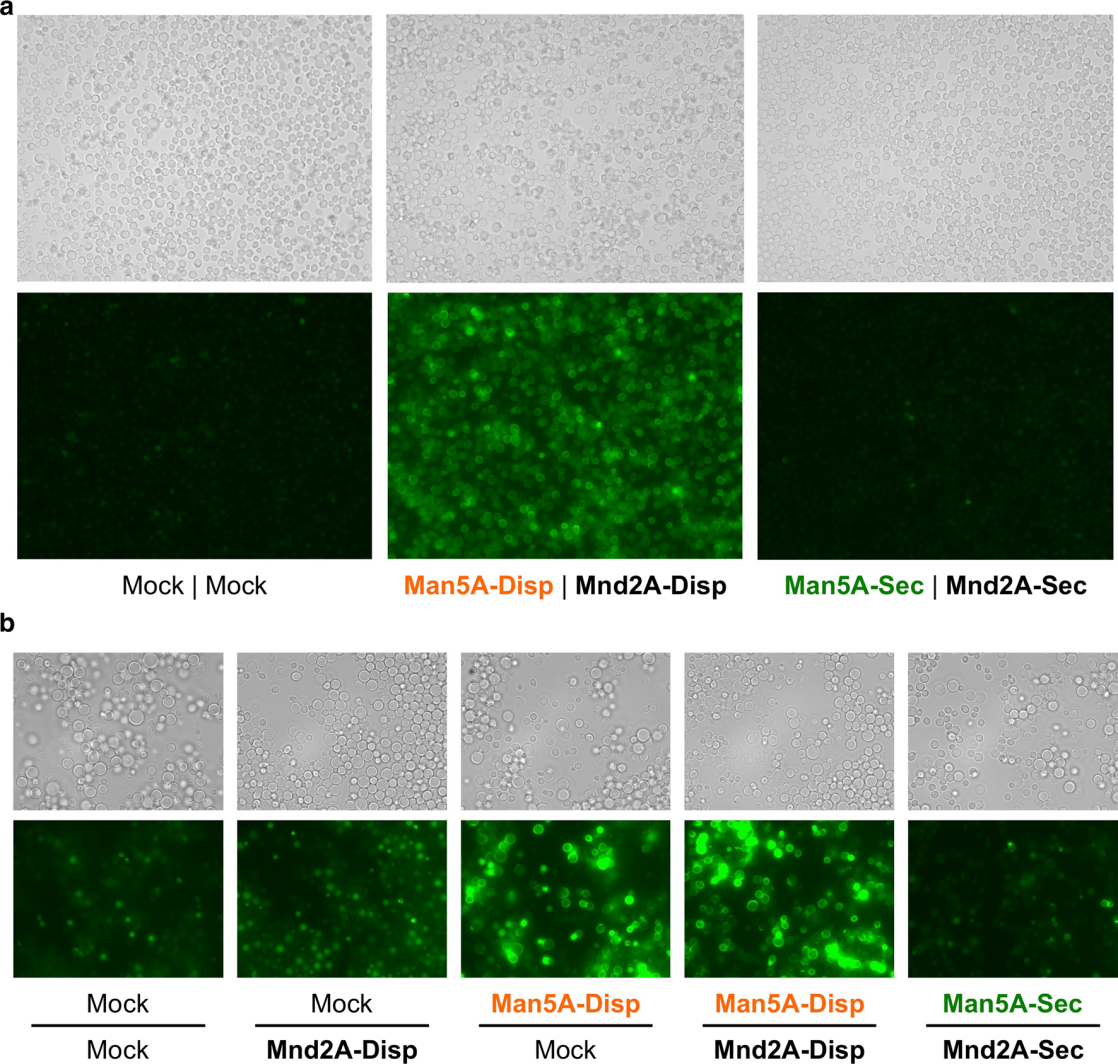
Fluorescence images of immunostained Man5A-displaying and -secreting yeast cells. **a**, **b** Yeast cells immunostained with Alexa Fluor 488-labeled anti-FLAG antibody. *Upper panels* show visible light images and *lower panels* show green fluorescence images. Images correspond to low magnification (×400) **(a)** and high magnification (×1000) **(b)**

Next, fluorescent immunostaining was performed to assess the presence of Man5A and Mnd2A on the yeast cell surface (Fig. 3; Additional file 1: Figure S2). Cultured yeast cells were stained with Alexa Fluor 488-labeled anti-FLAG and anti-HA antibodies. Immunostaining of Man5A- and Man5A/Mnd2A-displaying yeast cells with the anti-FLAG antibody revealed fluorescence along the outline of the yeast (that is, on the cell surface), whereas other cells did not exhibit obvious fluorescence (Fig. 3a, b). On the other hand, immunostaining of Mnd2A- and Man5A/Mnd2A-displaying yeast cells with the anti-HA antibody revealed weak fluorescence at levels that could clearly be discriminated from the absence of staining in other cells (Additional file 1: Figure S2). The relatively weaker fluorescence might reflect steric hindrance of binding to the anti-HA antibody or low display levels of the HA-tagged Mnd2A protein. However, given the results obtained with the β-mannosidase activity measurements (Fig. 2b), we considered it likely that the HA-tagged Mnd2A was being displayed on the yeast cell surface. Thus, we confirmed co-display on the yeast cell surface of β-mannanase (Man5A) and β-mannosidase (Mnd2A) from *A. aculeatus*, although the display of β-mannosidase might leave room for improvement in the future.

### Ethanol fermentation by *S. cerevisiae* using mannose as a carbon source

To evaluate the ability of *S. cerevisiae* to produce ethanol from mannose, three constructed yeast cells (Mock/Mock, Man5A-Disp/Mnd2A-Disp, and Man5A-Sec/Mnd2A-Sec) (Table 2) were cultured in media containing mannose as a sole carbon source (Additional file 1: Figure S3). Following pre-cultivation in SDC media, yeast cells were harvested and used to inoculate yeast extract–peptone–acetate (YPA) media (pH 5.0) containing 5 g/L mannose to a starting optical density at 600 nm of 20 (OD_600_ = 20). After 18 h of growth, ethanol concentrations in fermented media were measured. All three yeast strains produced ethanol at concentrations of 2.0~2.2 g/L (Additional file 1: Figure S3). Productivities of ethanol at 4 h were similar to those seen at 18 h (data not shown). These values were approximately 80 % of theoretical yield, suggesting that mannose fermentation in *S. cerevisiae* was practicable and might serve as a capable technology for ethanol production.

### Ethanol fermentation from 1,4-β-d-mannan using yeast co-displaying β-mannanase and β-mannosidase

**Fig. 4:**
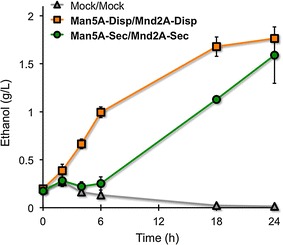
Ethanol fermentations from 1,4-β-d-mannan using Man5A- and Mnd2A-displaying and -secreting yeast cells. Fermentations were performed in YPA medium (pH 5.0) containing 5 g/L 1,4-β-d-mannan as a carbon source. Fermentations were started with an OD_600_ = 20 of yeast cells. Data are presented as the mean ± standard deviation of separate cultivations (*n* = 3 each)

To test the capability of β-mannanase- and β-mannosidase-co-displaying yeast cells for ethanol fermentation from mannan, three constructed yeast strains (Mock/Mock, Man5A-Disp/Mnd2A-Disp, and Man5A-Sec/Mnd2A-Sec) (Table 2) were cultured in media containing 1,4-β-d-mannan (Fig. 4). The 1,4-β-d-mannan was provided as galactosidase-treated carob galactomannan; this enzyme treatment removes essentially all of the α-linked d-galactosyl residues, rendering a substrate that contains 97 % mannose and 3 % galactose (based on the manufacturer’s analysis).

First, we measured the quantities of major sugar components in the 1,4-β-d-mannan using the standard biomass analytical method provided by the National Renewable Energy Laboratory (NREL) (Table 3). The contents of mannose and galactose contained in total sugars were almost coincident with the manufacture’s structure. The total amounts of mannan, galactose and glucose sugars accounted for 87.2 % (g/g) of the 1,4-β-d-mannan (Table 3).

**Table 3:** Major sugar components of mannans used in this study

Sugar components contained in biomass (%; g/g)						
	Mannose	Galactose	Glucose	Xylose	Arabinose	Total
1,4-β-d-Mannan	83.0 ± 4.0	2.1 ± 0.1	2.1 ± 0.1	ND	ND	87.2 ± 4.1
Ivory nut mannan	99.8 ± 1.5	1.0 ± 0.0	0.9 ± 0.0	ND	ND	101.6 ± 1.5

After pre-culturing in SDC media, cells were harvested and used to inoculate YPA media containing 5 g/L 1,4-β-d-mannan to a starting OD_600_ of 20. Mock/Mock cells that did not contain the β-mannanase and β-mannosidase genes produced only low levels of ethanol (Fig. 4). Similarly, Man5A-Sec/Mnd2A-Sec yeast cells, which secreted β-mannanase and β-mannosidase outside the cells, produced only low levels of ethanol up to 6 h of fermentation; subsequently, this strain gradually started to produce ethanol, achieving an ethanol titer of 1.590 ± 0.305 g/L after 24 h (Fig. 4). In contrast, Man5A-Disp/Mnd2A-Disp cells, which displayed β-mannanase and β-mannosidase on the cell surface, produced ethanol starting immediately after inoculation, achieving ethanol titers of 0.995 ± 0.188 and 1.764 ± 0.099 g/L after 6 and 24 h of fermentation, respectively (Fig. 4). Thus, Man5A-Disp/Mnd2A-Disp cells produced 79.3 % ethanol as theoretical yield from 5 g/L 1,4-β-d-mannan.

Additionally, we performed the 1,4-β-d-mannan fermentations with the addition of commercially available enzymes. The two strains (Man5A-Disp/Mnd2A-Disp and Man5A-Sec/Mnd2A-Sec) were pre-cultured as above, then used to inoculate YPA media containing 5 g/L 1,4-β-d-mannan, additionally supplemented with β-mannanase from *Cellvibrio japonicus* (500 U) and β-mannosidase from *Cellulomonas fimi* (8 U) (Table 2; Additional file 1: Figure S4). Both Man5A-Disp/Mnd2A-Disp and Man5A-Sec/Mnd2A-Sec produced ethanol without delay from the culture inoculation, achieving ethanol titers after 24 h of fermentation of 1.725 ± 0.039 and 1.720 ± 0.294 g/L, respectively (Additional file 1: Figure S4). Notably, these values were similar to that obtained with Man5A-Disp/Mnd2A-Disp grown for 24 h in medium not supplemented with exogenous enzymes (Fig. 4). In addition, YPA-rich (non-selection) medium was not harmful for the ethanol fermentation using the plasmid-introducing transformants, since it has been known that the yeast cells could moderately maintain the plasmid retention rates in YP-rich medium containing glucose (YPD medium) [28].

**Fig. 5:**
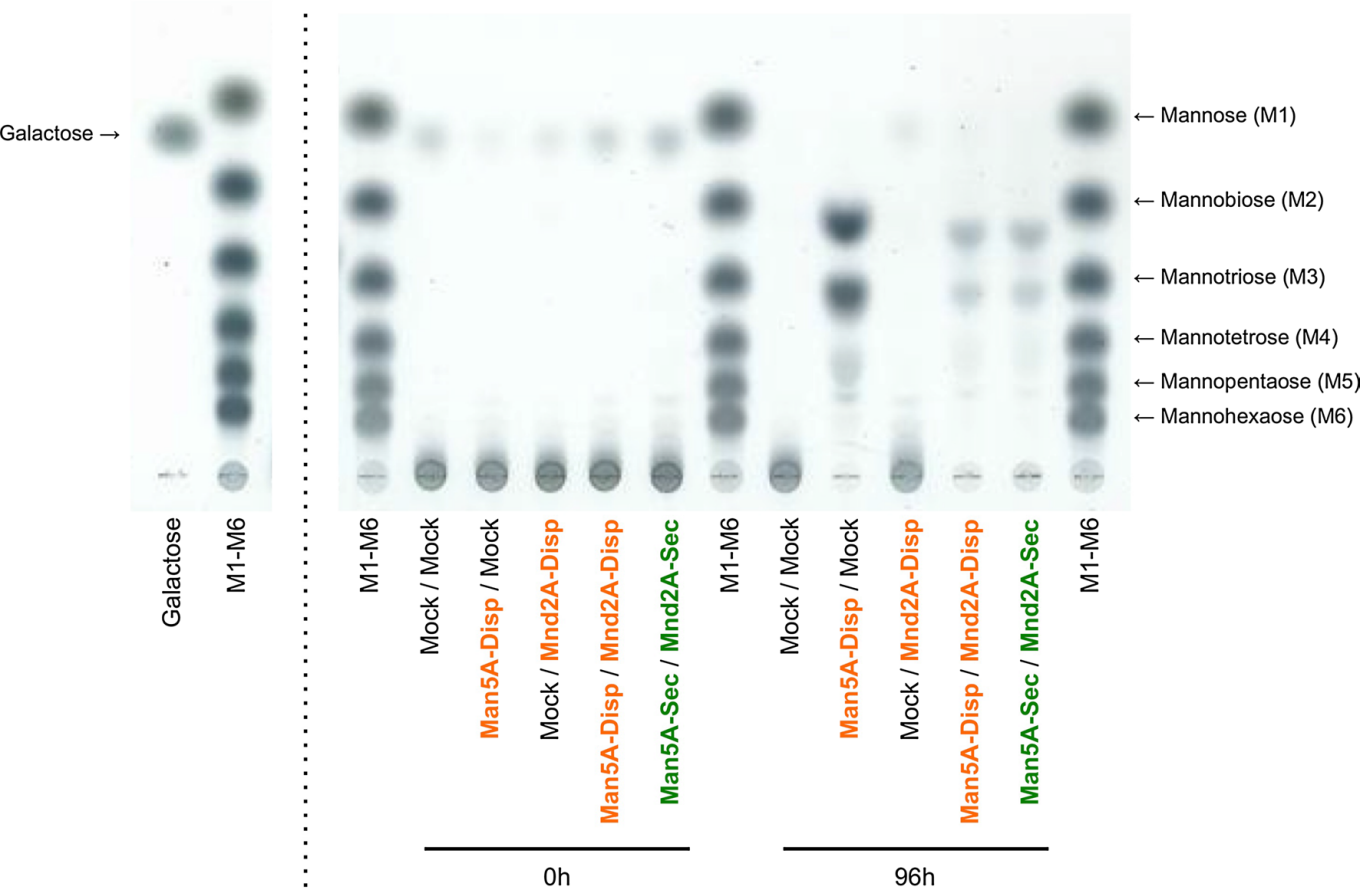
Residual products of 1,4-β-d-mannan fermentations. The residual products of 1,4-β-d-mannan fermentations using Man5A- and/or Mnd2A-displaying and -secreting yeast cells were analyzed by TLC. Galactose and manno-oligosaccharides (mannose to mannohexaose; M1–M6) were used as standards

To assess the residual products in the 1,4-β-d-mannan fermentations, thin-layer chromatography (TLC) was performed. To permit degradation by the expressed enzymes to achieve completion, the fermentations (in the media lacking exogenous purified enzymes) were extended to 96 h. At the beginning of fermentation (0 h), galactose was detected in the mannan-supplemented fermentation medium into which each of the tested strains was inoculated (Fig. 5). After 96 h, the Man5A-Disp strain exhibited two major spots in TLC, along with several minor (lower intensity) higher molecular weight spots (Fig. 5). The two major spots were assumed to correspond to disaccharide and trisaccharide, although the mobility of these spots appeared to differ slightly from those observed for unmodified mannobiose and mannotriose. We, therefore, postulated that these spots corresponded to modified (e.g., glucosylated, galactosylated or acetylated) mannosaccharides [10]. As to the higher molecular weight spots, we noted that these spots were observed following fermentation by the Man5A-Disp strain (i.e., cells not capable of expressing β-mannosidase). Therefore, we postulated that the higher molecular weight spots corresponded to longer manno-oligosaccharides with and without modifications (of unknown identity). In contrast, the higher molecular weight spots were effectively eliminated in cultures of the Man5A-Disp/Mnd2A-Disp and Man5A-Sec/Mnd2A-Sec strains; cultures of these strains also showed decreases in the intensities of the two major spots (Fig. 5). Thus, the degradation of the minor and major spots correlated with β-mannosidase expression. Together, these results suggested that the production of Man5A in yeast yielded di- or tri-manno-oligosaccharides, including oligosaccharides modified by unknown moieties. Additionally, Mnd2A appeared to act on several longer manno-oligosaccharides as well as mannobiose and mannotriose, including those associated with the unknown modification.

### Other applications of β-mannanase- and β-mannosidase-co-displaying yeast in mannan fermentations

**Fig. 6:**
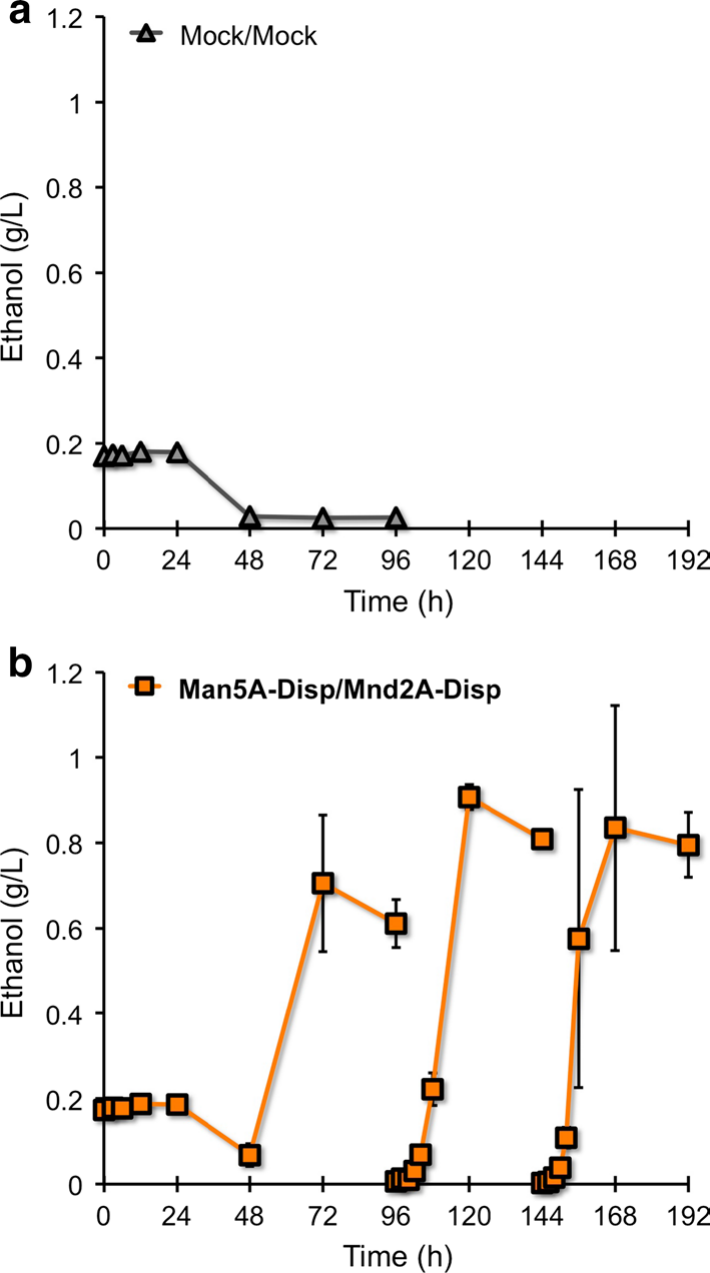
Repeated ethanol fermentation from 1,4-β-d-mannan using Man5A- and Mnd2A-displaying yeast cells. **a** Mock/Mock yeast cells. **b** Man5A-Disp/Mnd2A-Disp yeast cells. Fermentations were performed in SCA media (pH 5.0) containing 5 g/L 1,4-β-d-mannan as a carbon source. First batch fermentations were started with an OD_600_ = 0.05 of yeast cells. For second and third batch fermentations, the cells were collected, washed, and resuspended in fresh SCA media containing 5 g/L 1,4-β-d-mannan. Data are presented as the mean ± standard deviation of separate cultivations (*n* = 3 each)

To demonstrate recycling of β-mannanase- and β-mannosidase-co-displaying yeast cells, we tested repeated fermentation from 1,4-β-d-mannan (Fig. 6). To maintain the plasmids during the repeated fermentation, synthetic casamino acids–acetate (SCA) selection medium (pH 5.0) was used. As a first step, we tested whether our yeast strains could grow using 1,4-β-d-mannan as a sole carbon source. Specifically, cells were used to inoculate SCA media containing 5 g/L 1,4-β-d-mannan to give an initial OD_600_ of 0.05. Under these conditions, the Mock/Mock strain produced 0.180 ± 0.002 g/L ethanol after 24 h of growth; we presumed that this ethanol production reflected fermentation of the residual galactose contained in 1,4-β-d-mannan (the galactosidase-treated carob galactomannan). Subsequently, this ethanol was completely assimilated; minimal further growth was observed through 96 h of fermentation (Fig. 6a), and cultures of this strain were terminated after this first batch fermentation. Similarly, Man5A-Disp/Mnd2A-Disp yeast cells produced 0.188 ± 0.012 g/L ethanol by 24 h, a process again attributed to fermentation of the residual galactose in the substrate, and this ethanol was depleted by 48 h of culturing (Fig. 6b). However, in contrast to the Mock/Mock culture, the Man5A-Disp/Mnd2A-Disp cells subsequently grew, achieving a yield of 0.705 ± 0.159 g/L ethanol after 72 h (Fig. 6b). After 96 h, the cells of this culture were collected by centrifugation, washed, and used to inoculate fresh SCA medium containing 5 g/L 1,4-β-d-mannan. During this second batch fermentation, the cells produced ethanol from the time of inoculation (without a lag period), achieving an ethanol titer of 0.907 ± 0.029 g/L (40.8 % of theoretical yield) after 24 h in fresh medium (i.e., after a total of 120 h of growth) (Fig. 6b). After 48 h in fresh medium (total 144 h), the cells again were collected, washed, and used to inoculate fresh SCA medium containing 5 g/L 1,4-β-d-mannan. During this third batch fermentation, the cells produced ethanol [after 24 h in fresh medium (total 168 h)] to levels similar to those observed in the second batch fermentation (Fig. 6b). The lower titers of ethanol production during the second and third batch fermentations [compared to those obtained in the single batch fermentation (1.764 ± 0.099 g/L; 79.3 % of theoretical yield) in Fig. 4] might be attributed to the difference in the media or the relatively smaller initial cell densities used for subsequent batch fermentations (OD_600_ = 20 in Fig. 4). Nevertheless, these results indicated that the recovered Man5A-Disp/Mnd2A-Disp yeast cells exhibited β-mannanase and β-mannosidase activities from early stages in batch fermentations. We hypothesize that the recovered yeast cells were able to ferment starting from the initiation of subsequent batch cultures because the harvested cells already were displaying the relevant mannose-degrading enzymes. Thus, Man5A- and Mnd2A-co-displaying yeast cells possess demonstrable utility for repeated cycles of batch fermentation from 1,4-β-d-mannan.

**Fig. 7:**
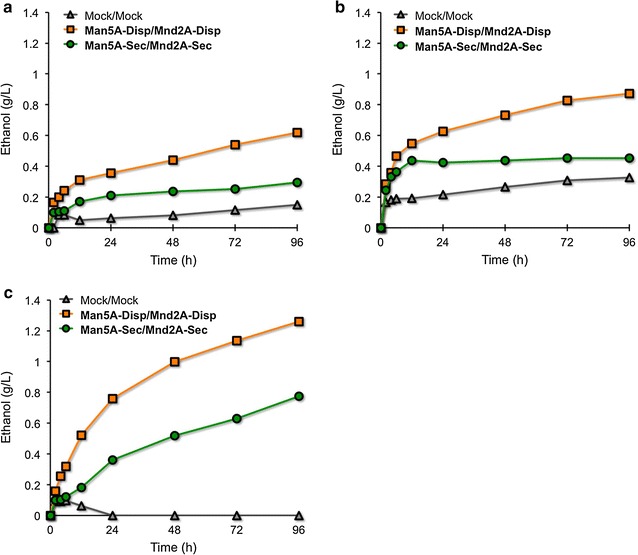
Ethanol fermentations from ivory nut mannan using Man5A- and Mnd2A-displaying and -secreting yeast cells. **a** Fermentation was performed in YPA medium (pH 5.0) containing 5 g/L ivory nut mannan as a carbon source. **b** Fermentation was performed in YPA medium (pH 5.0) containing 5 g/L ivory nut mannan as a carbon source, and 500 U β-mannanase and 8 U β-mannosidase as purified enzymes. **c** Fermentation was performed in YPA medium (pH 5.0) containing 5 g/L NaOH-treated ivory nut mannan as a carbon source. Culture was initiated with an OD_600_ = 20 of yeast cells

Additionally, we tested ethanol fermentation by our strain from an alternative biomass source, ivory nut mannan (Fig. 7). Following pre-culturing as above, the three yeast strains (Mock/Mock, Man5A-Disp/Mnd2A-Disp, and Man5A-Sec/Mnd2A-Sec) (Table 2) were used to inoculate YPA media containing 5 g/L ivory nut mannan to an initial OD_600_ of 20 (Fig. 7a). After 96 h of fermentation, Mock/Mock cells produced low levels of ethanol (0.149 g/L; 5.8 % of theoretical yield), while Man5A-Sec/Mnd2A-Sec cells produced ethanol at a twofold higher level (0.295 g/L; 11.4 % of theoretical yield). In contrast, after 96 h, Man5A-Disp/Mnd2A-Disp cells produced ethanol at an even higher level (0.618 g/L; 23.9 % of theoretical yield), although this titer was still only one-third of that obtained with 1,4-β-d-mannan as the substrate. Hence, we tested the fermentations in ivory nut mannan-containing media additionally supplemented with purified β-mannanase and β-mannosidase (Fig. 7b). All three strains exhibited slightly higher levels of ethanol compared to the respective cultures performed in media not supplemented with the purified enzymes, yielding (in the case of Man5A-Disp/Mnd2A-Disp cells) 0.871 g/L of ethanol (33.6 % of theoretical yield) after 96 h. Despite the presence of purified enzymes, the ethanol yield was not significantly changed. Therefore, we performed the fermentations using media containing pre-treated ivory nut mannan (without purified enzymes). Specifically, ivory nut mannan was processed with NaOH, neutralized, and then added at 5 g/L to YPA medium; the resulting medium was used for culturing of the same panel of 3 strains (Fig. 7c). Under these conditions, ethanol production by Mock/Mock cells was barely detectable, peaking at ~0.1 g/L at 8 h (~3.9 % of theoretical yield). Man5A-Sec/Mnd2A-Sec cells accumulated ethanol throughout the culturing interval, achieving a titer of 0.776 g/L after 96 h of fermentation (30.0 % of theoretical yield). In contrast, Man5A-Disp/Mnd2A-Disp cells exhibited a more rapid kinetics of ethanol accumulation, achieving a titer of 1.260 g/L of ethanol after 96 h (48.6 % of theoretical yield). Thus, we proved that ethanol fermentation from ivory nut mannan was feasible using Man5A- and Mnd2A-co-displaying yeast cells; base treatment of this substrate provided an approximately twofold increase in ethanol yield.

Finally, we tested the fermentations from 100 g/L 1,4-β-d-mannan and NaOH-treated ivory nut mannan using the Man5A-Disp/Mnd2A-Disp yeast strain (harboring pFGK426-AaMan5A and pFGK424-AaMnd2A; autonomous replicating 2*μ* multi-copy plasmids). To consider the retention manners of mannanase- and mannosidase-displaying genes in addition to the high concentrations of mannan substrates, the genomic integration plasmids were newly constructed (pFGK406-AaMan5A and pFGK402-AaMnd2A) (Table 1). The constructed plasmids were integrated into the *ura3* and *ade2* genomic loci in YPH499 (Man5A-Disp-G/Mnd2A-Disp-G) (Table 2).

**Fig. 8:**
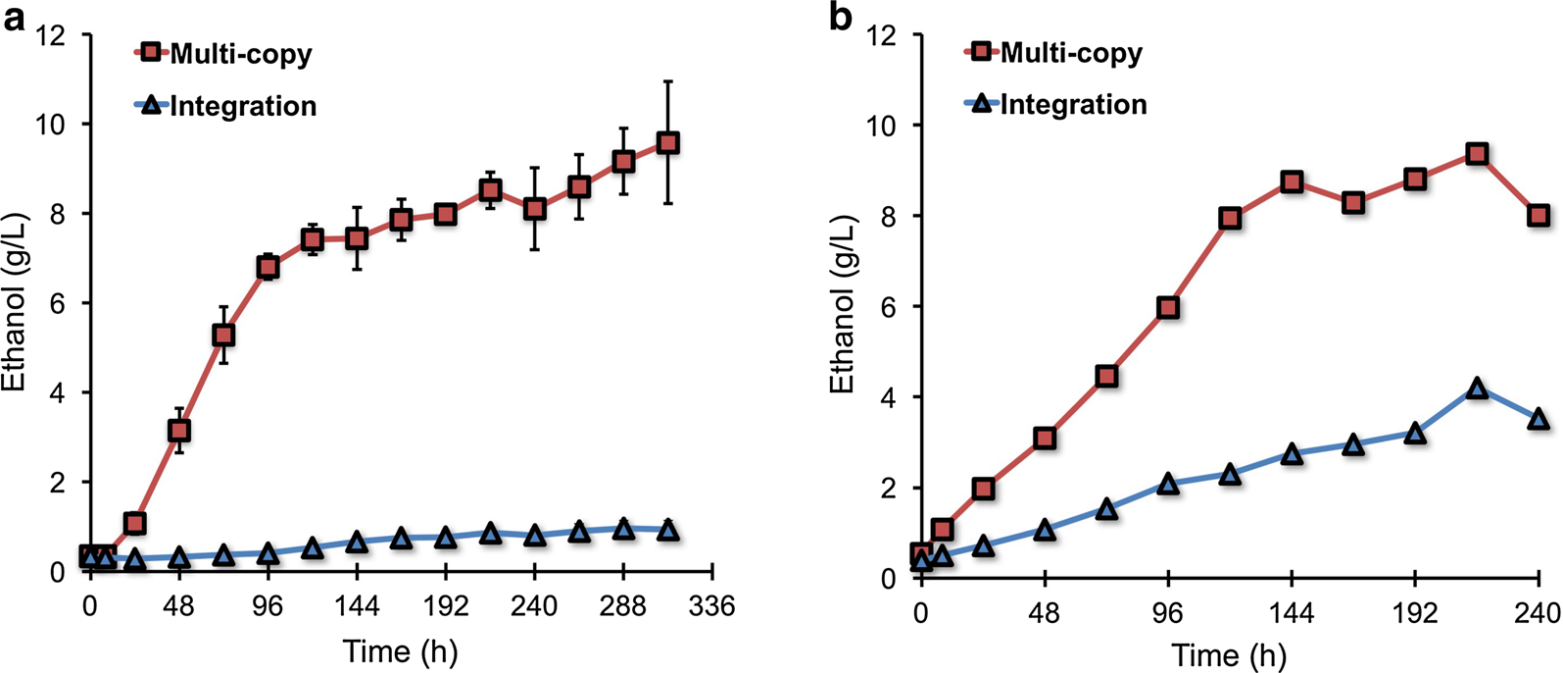
Ethanol fermentations from 100 g/L 1,4-β-d-mannan and ivory nut mannan using Man5A- and Mnd2A-displaying yeast cells. **a** Fermentations were performed in YPA media (pH 5.0) containing 100 g/L 1,4-β-d-mannan as a carbon source. Data are presented as the mean ± standard deviation of separate cultivations (*n* = 3 each). **b** Fermentations were performed in YPA media (pH 5.0) containing 100 g/L NaOH-treated ivory nut mannan as a carbon source. Fermentations were started with an OD_600_ = 20 of yeast cells. Multi-copy (Man5A-Disp/Mnd2A-Disp): YPH499 harboring pFGK426-AaMan5A and pFGK424-AaMnd2A (multi-copy 2μ plasmids). Integration (Man5A-Disp-G/Mnd2A-Disp-G): YPH499 integrating pFGK406-AaMan5A and pFGK402-AaMnd2A (genomic integration plasmids)

For 100 g/L 1,4-β-d-mannan fermentations, Man5A-Disp/Mnd2A-Disp yeast cells (multi-copy) produced 6.809 ± 0.283 and 9.579 ± 1.362 g/L ethanol (15.3 and 21.5 % of theoretical yields), respectively, at 96 and 312 h, whereas Man5A-Disp-G/Mnd2A-Disp-G yeast cells (integration) marginally produced ethanol after 312 h (0.936 ± 0.199 g/L; 2.1 % of theoretical yield) (Fig. 8a). In contrast, when using 100 g/L NaOH-treated ivory nut mannan for fermentations, the multi-copy yeast cells produced 7.951 and 9.362 g/L ethanol (15.3 and 18.1 % of theoretical yields), respectively, at 120 and 216 h, while the integration yeast cells produced 4.209 g/L ethanol after 216 h (8.1 % of theoretical yield) (Fig. 8b). These results imply that the multi-copy plasmids favorably operated to exhibit higher mannan-degrading activities than the genomic integration plasmids, although there remains the issue related to the persistence of plasmid retentions. It should improve the ethanol yields in the future; however, we thus succeeded in the demonstration of ethanol fermentations from high concentration mannans using mannanase- and mannosidase-co-displaying yeast cells.

## Conclusions

In this study, we constructed a recombinant yeast strain that co-displayed β-mannanase and β-mannosidase on its cell surface. The parental strain could assimilate monomeric mannose to produce ethanol at levels similar to those obtained by fermentation of glucose. The constructed yeast strain exhibited β-mannanase and β-mannosidase activities, although the display of β-mannosidase might be improved in the future.

The constructed β-mannanase- and β-mannosidase-co-displaying yeast cells hydrolyzed 1,4-β-d-mannan and produced ethanol by assimilating the degraded sugars. The constructed yeast cells also enabled repeated batch fermentation from 1,4-β-d-mannan, and the yeast cells recovered after the second batch fermentation showed the ability to degrade 1,4-β-d-mannan from the early stages of the batch fermentations (i.e., without an apparent lag phase). These cells produced ethanol by fermentation from both non-treated and NaOH-treated ivory nut mannan. In addition, they succeeded in the direct degradation and fermentation from 100 g/L 1,4-β-d-mannan and NaOH-treated ivory nut mannan. Thus, we demonstrated the capability and utility of the β-mannanase- and β-mannosidase-co-displaying yeast cells for ethanol fermentations from linear mannans.

Yeast has several advantages for biorefining, including the potential for bulk-scale production of fermentation compounds. Yeast is also tolerant to low pH and robust against autolysis, thereby reducing the risk of contamination and permitting long-term repeated or continuous fermentation. These attributes are expected to contribute to cost reduction and to facilitate commercial viability [29]. Therefore, the co-displaying of β-mannanase and β-mannosidase on the yeast cell surface is a promising strategy applicable to yeast fermentations designed to produce other (non-ethanol) biofuels and biochemicals in metabolically engineered *S. cerevisiae* [30–32]. In future studies, the additional display of other enzymes (such as glucosidase and galactosidase) on the mannanase- and mannosidase-co-displaying yeast cells is expected to enable the fermentation of glucomannans and galactoglucomannans. In combination with cellulase and xylanase, our approach promises to be a powerful platform technology for yeast fermentation from lignocellulosic materials including those that incorporate mannan components, as well as mannan carbohydrate biomass.

## Methods

### Yeast strain and media

*Saccharomyces cerevisiae* YPH499 (*MATa ura3*-*52 lys2*-*801 ade2*-*101 trp1*-Δ*63 his3*-Δ*200 leu2*-Δ*1*) [33] (Stratagene/Agilent Technologies, Palo Alto, CA, USA) was used as the host yeast strain. SD medium contained 6.7 g/L yeast nitrogen base without amino acids (YNB) (BD-Diagnostic Systems, Sparks, MD, USA) and 20 g/L glucose. SM medium contained 6.7 g/L YNB and 20 g/L mannose. For SDC medium, 20 g/L casamino acids (BD-Diagnostic Systems) was added to SD medium. YPA medium contained 10 g/L yeast extract (Nacalai Tesque, Kyoto, Japan), 20 g/L peptone (BD-Diagnostic Systems), and 200 mM sodium acetate (Nacalai Tesque), and the pH was adjusted to 5.0. SCA medium contained 6.7 g/L YNB, 20 g/L casamino acids, and 200 mM sodium acetate, and the pH was adjusted to 5.0. Amino acids and nucleotides (40 mg/L adenine, 20 mg/L histidine, 60 mg/L leucine, 20 mg/L lysine, 40 mg/L tryptophan, and 20 mg/L uracil) were included in each medium (except YPA) to provide the relevant auxotrophic components. Briefly, all six amino acids and nucleotides were supplemented for minimal media, and four amino acids and nucleotides lacking tryptophan and uracil (adenine, histidine, leucine and lysine) or adenine and uracil (histidine, leucine, lysine and tryptophan) were supplemented for selection media.

### Plasmid construction

All plasmids and primers used in this study are listed in Table 1; Additional file 1: Table S1, respectively.

A DNA fragment containing the sequences coding for the secretion signal of α-factor and the Flo428 anchor protein (C-terminal 428 a.a. of Flo1p) was amplified from pFGK426 [26] and then digested with *Nhe*I and *Bam*HI. The amplified fragment [that contains a multi-cloning site (*Sal*I, *Sph*I, and *Pac*I) between the two coding sequences] was ligated into *Nhe*I, *Bgl*II double-digested pGK424 [28], resulting in pFGK424.

The ORFs encoding FLAG-fused β-mannanase, Man5A (alias name: Man1) [14, 15], without and with stop codons, were amplified from reverse-transcribed cDNA of *A. aculeatus* NBRC 5330 [NBRC; National Institute of Technology and Evaluation (NITE) Biological Resource Center]; these ORFs correspond to accession number L35487. The amplified *man5A* ORFs (without and with stop codons) were inserted into the *Sal*I-digested pFGK426 vector, which was derived from pGK426 [28], using In-Fusion HD Cloning Kit (Clontech Laboratories/Takara Bio, Shiga, Japan), yielding, respectively, pFGK426-AaMan5A (for display) and pFGK426-AaMan5A-TAA (for secretion).

The genes encoding HA-fused β-mannosidase, Mnd2A (alias name: ManB) [18], without and with stop codons, were, respectively, amplified from reverse-transcribed cDNA of *A. aculeatus* NBRC 5330; these ORFs correspond to accession number AB015509. The amplified *mnd2A* genes were inserted into the *Sal*I-digested pFGK424 vector using In-Fusion HD cloning kit, yielding, respectively, pFGK424-AaMnd2A (for display) and pFGK424-AaMnd2A-TAA (for secretion).

The DNA fragments containing expression cassettes for displaying Man5A and Mnd2A on yeast cell surface were, respectively, prepared by digesting pFGK426-AaMan5A and pFGK424-AaMnd2A with *Nco*I/*Sac*I and *Apa*I/*Bsa*I. They were ligated with the pRS406 and pRS402 vectors (ATCC; American Type Culture Collection) digested at the same sites, generating pFGK406-AaMan5A and pFGK402-AaMnd2A integration vectors.

### Yeast transformation

Transformation was carried out using the lithium acetate method [34]. YPH499 was co-transformed with pFGK426 and pFGK424 or the constructed plasmids. For genomic integration, pFGK406-AaMan5A and pFGK402-AaMnd2A were digested on single-cut sites within the marker sequences, and were used to transform YPH499. All transformants generated in this study are listed in Table 2.

### Growth tests

YPH499 was grown overnight in SD minimal medium at 30 °C and then harvested. After washing, the cells were inoculated into 5 mL of SD or SM minimal medium to give an OD_660_ = 0.05. Then, the cells were cultivated at 30 °C with shaking at 70 rpm for up to 48 h, and the OD_660_ (cell growth) was automatically monitored every 30 min using a TVS062CA biophotorecorder (Advantec Toyo, Tokyo, Japan). Specific growth rates (μ) were calculated at 6–8 h.

### Enzyme assays

The transformants were grown overnight in SD selection media at 30 °C and then inoculated into 5 mL of SDC selection medium to give an OD_600_ = 0.05. Cells then were grown at 30 °C at 150 rpm for 24 h and the OD_600_ of the culture media was measured. Cells were collected (by centrifuging the cultures for 3 min at 3000×*g*), washed, and then concentrated tenfold by resuspending in 500 μL of distilled water.

Relative β-mannanase activity was measured by monitoring the absorbance of azo dye released from azo-carob galactomannan (Megazyme, Wicklow, Ireland). Reaction mixtures containing 60 μL of 2 % azo-carob galactomannan, 12 μL of 500 mM sodium acetate (pH 5.0), and 48 μL of the cell suspensions were incubated at 45 °C for 30 min. Then, 300 μL of 95 % ethanol was added, the mixture was vortexed for 10 s, and the samples were cooled on ice for 10 min and centrifuged for 10 min at 10,000×*g* at room temperature. The resulting supernatants were transferred to 96-well microplates and the absorbance at 590 nm (ABS_590_) was measured using an EnVision multilabel plate reader (Perkin-Elmer, Waltham, MA, USA). Relative β-mannanase activities were determined as the ABS_590_ values normalized per OD_600_ unit.

β-Mannosidase activity was measured by determining the amount of *p*-nitrophenol (*p*NP) released from *p*NP-mannopyranoside (Megazyme). Reaction mixtures containing 40 μL of 3 mM *p*NP-mannopyranoside, 12 μL of 500 mM sodium acetate (pH 5.0), and 68 μL of the cell suspensions were incubated at 45 °C for 30 min. Then, 120 μL of 1 M sodium carbonate was added, the mixture was vortexed for 10 s, and the samples were centrifuged for 10 min at 10,000×*g* at room temperature. The resulting supernatants were transferred to 96-well microplates and the ABS_405_ was measured using an EnVision multilabel plate reader. One unit of enzyme activity was defined as the amount of enzyme that liberated 1 μmol of *p*NP per min under the above conditions. β-Mannosidase activities were determined as units normalized per OD_600_ unit.

### Immunofluorescence staining

The transformants were grown overnight in SD selection media at 30 °C and then inoculated into 30 mL of SDC selection media additionally supplemented with 110 mg/L adenine (to avoid the autofluorescence derived from the pink-colored precursor of adenine biosynthesis) to give an OD_600_ = 0.05. Yeast cells then were grown at 30 °C at 150 rpm for 72 h. Cells were collected (by centrifuging the cultures for 2 min at 5000×*g*), washed, and then resuspended in phosphate-buffered saline (PBS) to OD_600_ = 20. An aliquot (200 μL) of the cell suspension was combined with 2 μL of Alexa Fluor 488-labeled anti-DDDDK-tag antibody (anti-FLAG antibody) (Medical & Biological Laboratories, Nagoya, Japan) or anti-HA-tag antibody (Covance, Princeton, NJ, USA). After incubation at 4 °C for 1 h, the cells were washed three times, resuspended in 50 μL of PBS, and observed using a BZ-9000 fluorescent microscope (Keyence, Osaka, Japan). Fluorescence images were acquired with a GFP-BP filter (ex. 470/40 nm, em. 535/50 nm).

### Ethanol fermentations

The transformants were grown overnight in SD selection media at 30 °C and then inoculated into 400 mL of SDC selection media to give an OD_600_ = 0.05. After cultivation at 30 °C at 150 rpm for 72 h, the yeast cells were harvested and washed three times. For mannose fermentations, the cells were inoculated into 20 mL of YPA media containing 5 g/L mannose as a sole carbon source to give an OD_600_ = 20, and cultivation was initiated.

For these (and subsequent) fermentations, cultivations were performed using a PPS-2511 ChemiStation (Tokyo Rikakikai, Tokyo, Japan) at 30 °C under oxygen-limited conditions; cells were grown with mild agitation (120 rpm) in closed glass tubes equipped with a filter-tipped CO_2_ outlet.

For 1,4-β-d-mannan fermentations, the collected cells were inoculated into 20 mL of YPA media containing 5 g/L 1,4-β-d-mannan (from carob galactomannan) (Megazyme) as a carbon source to give an OD_600_ = 20 and cultivation was initiated. For the equivalent fermentations in the presence of purified enzymes, β-mannanase from *Cellvibrio japonicus* (500 U) (Megazyme) and β-mannosidase from *Cellulomonas fimi* (8 U) (Megazyme) were added into YPA fermentation media containing 5 g/L 1,4-β-d-mannan, and cultivation was initiated as above.

For ivory nut mannan fermentations, the collected cells were inoculated into 20 mL of YPA media containing 5 g/L ivory nut mannan (Megazyme) as a carbon source to give an OD_600_ = 20, and cultivation was initiated as above. For the equivalent fermentations in the presence of purified enzymes, β-mannanase (500 U) and β-mannosidase (8 U) were added into YPA fermentation media containing 5 g/L ivory nut mannan. For pre-treated ivory nut mannan fermentations, 0.1 g of ivory nut mannan was suspended in 3 mL of distilled water and sonicated with an Ultrasonic Disruptor (UD-201; Tomy Seiko, Tokyo, Japan) for 15 min. Sodium hydroxide (1 mL of 10 N NaOH) was added to the sonicated mannan and the mixture was incubated for 3 h at room temperature. The mixture then was placed on ice and 10 N sulfuric acid (H_2_SO_4_) was gradually added to neutralize the suspension; the final volume was brought to 5 mL with distilled water. The NaOH-treated ivory nut mannan then was added to YPA fermentation media to a final concentration of 5 g/L (without purified enzymes).

For 1,4-β-d-mannan repeated fermentations, the transformants were grown overnight in SD selection media at 30 °C, and then inoculated into 20 mL of SCA selection media (pH 5.0) containing 5 g/L 1,4-β-d-mannan to give an OD_600_ = 0.05, and cultivation was initiated as above. After 96 h of fermentation, the yeast cells were harvested and washed three times. For the second batch fermentation, the collected cells were resuspended in 20 mL of fresh SCA media containing 5 g/L 1,4-β-d-mannan, and cultivation was initiated. After 48 h of this second fermentation, the yeast cells were harvested again and washed three times. For third batch fermentation, the collected cells were resuspended in 20 mL of fresh SCA media containing 5 g/L 1,4-β-d-mannan, and cultivation was initiated.

For fermentations of high-concentration mannans, the transformants were grown overnight in SD selection media at 30 °C, and then inoculated into 100 mL of SDC selection media to give an OD_600_ = 0.05. After cultivation at 30 °C at 150 rpm for 72 h, the yeast cells were harvested and washed three times. The collected cells were inoculated into 5 mL of YPA media containing 100 g/L 1,4-β-d-mannan and NaOH-treated ivory nut mannan to give an OD_600_ = 20, and cultivation was initiated using small anaerobic fermentation bottle with carbon dioxide (CO_2_) gas outlet.

### Analytical procedures

The concentrations of ethanol in the fermentation media were determined by following a previously described procedure using a GCMS-QP2010 Plus gas chromatography mass spectrometer (Shimadzu, Kyoto, Japan) equipped with a DB-FFAP column (Agilent Technologies) [35]. The quantities of major sugar components contained in 1,4-β-d-mannan and ivory nut mannan were determined by following a previously described procedure [36] according to the NREL method [37].

### Thin-layer chromatography (TLC)

The residual products in the 1,4-β-d-mannan fermentations were analyzed by TLC using a silica gel 60 plate (Merck, Darmstadt, Germany) developed in a solvent system consisting of *n*-butanol/acetic acid/water at a ratio of 2:1:1 (vol/vol) with galactose and several manno-oligosaccharides (mannose to mannohexaose) as standards. After development, the TLC plate was sprayed with diphenylamine/aniline/phosphate reagent [38] and heated at 120 °C for 10 min to visualize the digestion products.

## Additional file

10.1186/s13068-016-0600-4 Growth curves of YPH499 yeast strain in SD and SM media. YPH499 yeast cells were inoculated at an OD_660_ of 0.05 and cultured in SD and SM minimal media containing 2 g/L of glucose and mannose, respectively. The cell growth was determined by monitoring OD_660_ every 30 min using a TVS062CA biophotorecorder. Data are presented as the mean ± standard deviation of separate cultivations (*n* = 3 each). **Figure S2.** Fluorescence images of immunostained Mnd2A-displaying and -secreting yeast cells. Yeast cells immunostained with Alexa Fluor 488-labeled anti-HA antibody. Upper panels show visible light images and lower panels show green fluorescence images. Images correspond to high magnification (×1000). **Figure S3.** Ethanol fermentations by yeast cells using mannose as a carbon source. Fermentations were performed in YPA medium (pH 5.0) containing 5 g/L mannose as a sole carbon source. Fermentations were started with an OD_600_ = 20 of yeast cells. The ethanol titers show the values at 18 h of fermentations. Data are presented as the mean ± standard deviation of separate cultivations (*n* = 3 each). **Figure S4.** Ethanol fermentation by Man5A- and Mnd2A-displaying and -secreting yeast cells in 1,4-β-d-mannan-containing medium supplemented with purified enzymes. Fermentation was performed in YPA medium (pH 5.0) containing 5 g/L 1,4-β-d-mannan as a carbon source, and 500 U β-mannanase and 8 U β-mannosidase as purified enzymes. Cultures were initiated with an OD_600_ = 20 of yeast cells. Data are presented as the mean ± standard deviation of separate cultivations (*n* = 3 each). **Table S1.** List of primers.

## Abbreviations

ABS: absorbance
ATCC: American Type Culture Collection
CO_2_: carbon dioxide
GPI: glycosylphosphatidylinositol
NITE: National Institute of Technology and Evaluation
NBRC: National Institute of Technology and Evaluation Biological Resource Center
NREL: National Renewable Energy Laboratory
OD: optical density
ORF: open reading frame
PBS: phosphate-buffered saline
*p*NP-mannopyranoside: *p*-nitrophenyl-β-d-mannopyranoside
SCA: synthetic casamino acids–acetate
SD: synthetic dextrose
SM: synthetic mannose
SDC: synthetic dextrose–casamino acids
SSF: simultaneous saccharification and fermentation
TLC: thin-layer chromatography
YNB: yeast nitrogen base without amino acids
YPA: yeast extract–peptone–acetate
