# Supplementary material for: From mannan to bioethanol: cell surface co-display of β-mannanase and β-mannosidase on yeast Saccharomyces cerevisiae
Source: Biotechnol Biofuels. 2016 Sep 2;9(1):188. doi: 10.1186/s13068-016-0600-4 (PMC5009545; doi:10.1186/s13068-016-0600-4)
Supplement: Supplementary file 1 — 10.1186/s13068-016-0600-4 Growth curves of YPH499 yeast strain in SD and SM media. YPH499 yeast cells were inoculated at an OD660 of 0.05 and cultured in SD and SM minimal media containing 2 g/L of glucose and mannose, respectively. The cell growth was determined by monitoring OD660 every 30 min using a TVS062CA biophotorecorder. Data are presented as the mean ± standard deviation of separate cultivations (n = 3 each). Figure S2. Fluorescence images of immunostained Mnd2A-displaying and -secreting yeast cells. Yeast cells immunostained with Alexa Fluor 488-labeled anti-HA antibody. Upper panels show visible light images and lower panels show green fluorescence images. Images correspond to high magnification (×1000). Figure S3. Ethanol fermentations by yeast cells using mannose as a carbon source. Fermentations were performed in YPA medium (pH 5.0) containing 5 g/L mannose as a sole carbon source. Fermentations were started with an OD600 = 20 of yeast cells. The ethanol titers show the values at 18 h of fermentations. Data are presented as the mean ± standard deviation of separate cultivations (n = 3 each). Figure S4. Ethanol fermentation by Man5A- and Mnd2A-displaying and -secreting yeast cells in 1,4-β-d-mannan-containing medium supplemented with purified enzymes. Fermentation was performed in YPA medium (pH 5.0) containing 5 g/L 1,4-β-d-mannan as a carbon source, and 500 U β-mannanase and 8 U β-mannosidase as purified enzymes. Cultures were initiated with an OD600 = 20 of yeast cells. Data are presented as the mean ± standard deviation of separate cultivations (n = 3 each). Table S1. List of primers. [file 13068_2016_600_MOESM1_ESM.pdf]

Biotechnology for Biofuels

Additional file 1:

From mannan to bioethanol: cell surface co-display of β-mannanase and β-mannosidase on yeast *Saccharomyces cerevisiae*

Jun Ishii[1,†], Fumiyoshi Okazaki[2,†,§], Apridah Cameliawati Djohan[3], Kiyotaka Y Hara[2,¶], Nanami Asai-Nakashima[1], Hiroshi Teramura[1], Ade Andriani[3], Masahiro Tominaga[1], Satoshi Wakai[1], Prihardi Kahar[4], Yopi[3], Bambang Prasetya[3], Chiaki Ogino[4,*], Akihiko Kondo[1,4,5]

[1] Graduate School of Science, Technology and Innovation, Kobe University, Kobe, Japan

[2] Organization of Advanced Science and Technology, Kobe University, 1-1 Rokkodai, Nada, Kobe 657-8501, Japan

[3] Research Center for Biotechnology, Indonesian Institute of Sciences, Cibinong, Indonesia

[4] Department of Chemical Science and Engineering, Graduate School of Engineering, Kobe University, Kobe, Japan

[5] RIKEN Center for Sustainable Resource Science, Yokohama, Japan

[*] Correspondence: ochiaki@port.kobe-u.ac.jp

[†] Equal contributors

[§] Present address: Department of Life Sciences, Graduate School of Bioresources, Mie University, 1577 Kurimamachiya, Tsu, Mie 514-8507, Japan

[¶§] Present address: Department of Environmental Sciences, Graduate School of Nutritional and Environmental Sciences, University of Shizuoka, Shizuoka, Japan.

From mannan to bioethanol: cell surface co-displays of β-mannanase and β-mannosidase on yeast *Saccharomyces cerevisiae*

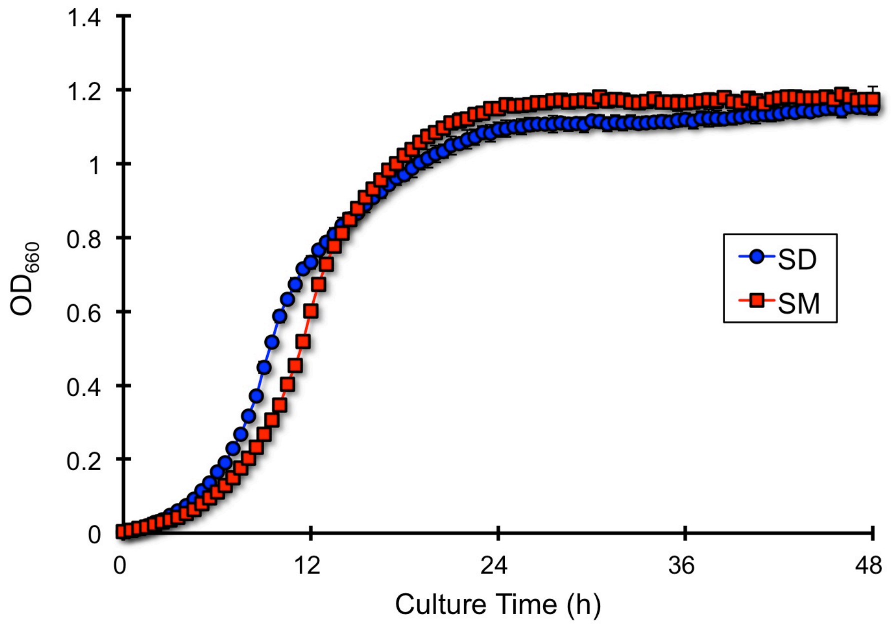

Figure S1. Growth curves of YPH499 yeast strain in SD and SM media. YPH499 yeast cells were inoculated at an OD_{660} of 0.05 and cultured in SD and SM minimal media containing 2 g/L of glucose and mannose, respectively. The cell growth was determined by monitoring OD_{660} every 30 min using a TVS062CA biophotorecorder. Data are presented as the mean ± standard deviation of separate cultivations (*n* = 3 each).

From mannan to bioethanol: cell surface co-displays of β-mannanase and β-mannosidase on yeast *Saccharomyces cerevisiae*

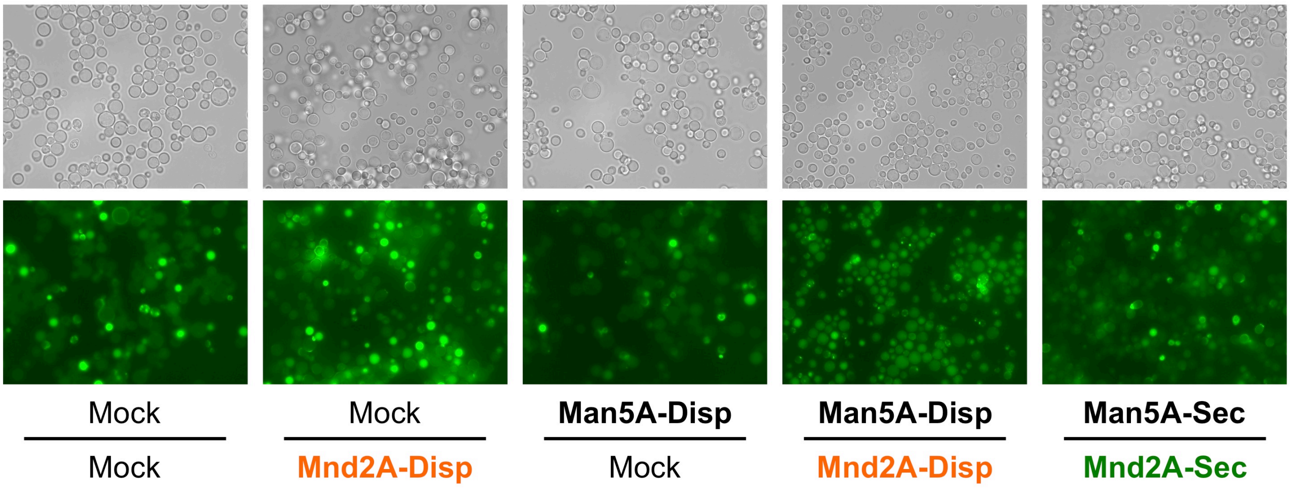

Figure S2. Fluorescence images of immunostained Mnd2A-displaying and -secreting yeast cells. Yeast cells immunostained with Alexa Fluor 488-labeled anti-HA antibody. Upper panels show visible light images and lower panels show green fluorescence images. Images correspond to high magnification (×1,000).

From mannan to bioethanol: cell surface co-displays of β-mannanase and β-mannosidase on yeast *Saccharomyces cerevisiae*

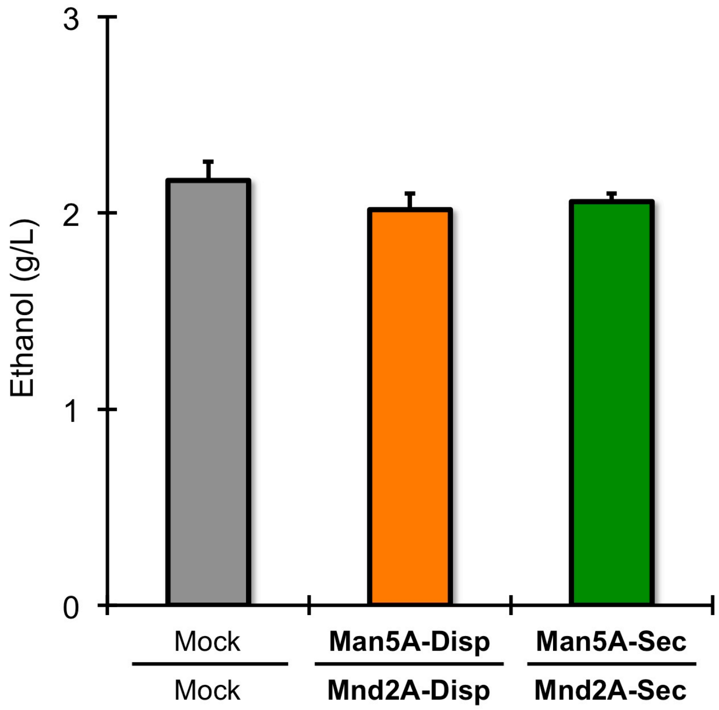

Figure S3. Ethanol fermentations by yeast cells using mannose as a carbon source. Fermentations were performed in YPA medium (pH 5.0) containing 5 g/L mannose as a sole carbon source. Fermentations were started with an OD_{600}=20 of yeast cells. The ethanol titers show the values at 18 h of fermentations. Data are presented as the mean ± standard deviation of separate cultivations (*n* = 3 each).

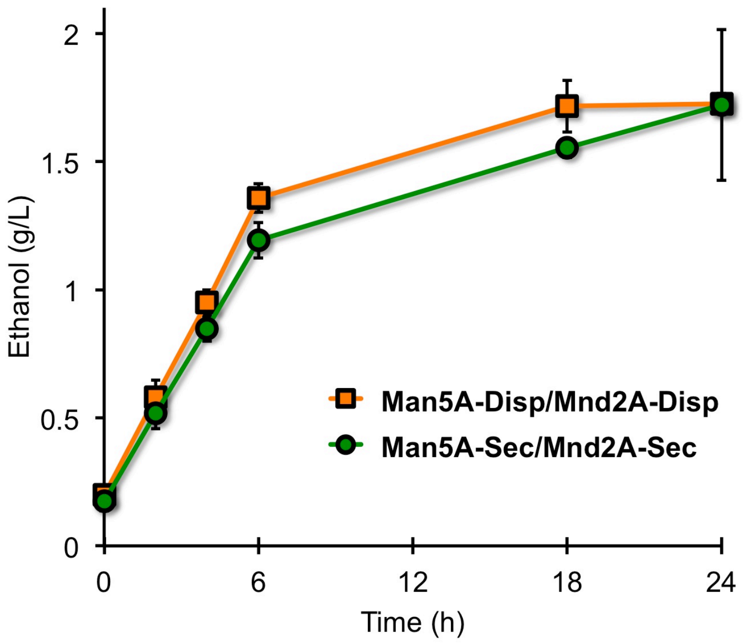

Figure S4. Ethanol fermentation by Man5A- and Mnd2A-displaying and -secreting yeast cells in 1,4-β-D-mannan-containing medium supplemented with purified enzymes. Fermentation was performed in YPA medium (pH 5.0) containing 5 g/L 1,4-β-D-mannan as a carbon source, and 500 U β-mannanase and 8 U β-mannosidase as purified enzymes. Cultures were initiated with an OD_{600}=20 of yeast cells. Data are presented as the mean ± standard deviation of separate cultivations (*n* = 3 each).

Table S1. List of primers

Construction of pFGK424

Amplification of the coding sequences for secretion signal sequence of α-factor and FLO428 anchor

5'–GGGGGCTAGCATGAGATTTCCTTCAATTTT

5'–GGGGGGATCCTTAAATAATTGCCAGCAAT

Construction of pFGK426-AaMan5A

Amplification of man5A-FLAG *(w/o TAA) gene*

5'–TAAAAGAGACGTCGACCTTCCCCGGACGCCGAACCACAAC

5'–TTAAGCATGCGTCGACCTTGTCATCGTCATCCTTGTAGTCCTTCGACTGCG CATTGATGGCCGCCAC

Construction of pFGK426-AaMan5A-TAA

Amplification of man5A-FLAG *(w/ TAA) gene*

5'–TAAAAGAGACGTCGACCTTCCCCGGACGCCGAACCACAAC

5'–TTAAGCATGCGTCGACTTACTTGTCATCGTCATCCTTGTAGTCCTTCGACT GCGCATTGATGGCCGCCAC

Construction of pFGK424-AaMnd2A

Amplification of mnd2A-HA *(w/o TAA) gene*

5'–TAAAAGAGACGTCGACCAATATGTTCGTGACCTGGGTACTGAAC

5'–TTAAGCATGCGTCGACAGCGTAATCTGGAACATCGTATGGGTATTCTCCCT CATTCAGATCCCACAG

Construction of pFGK424-AaMnd2A-TAA

Amplification of mnd2A-HA *(w/ TAA) gene*

5'–TAAAAGAGACGTCGACCAATATGTTCGTGACCTGGGTACTGAAC

5'–TTAAGCATGCGTCGACTTAAGCGTAATCTGGAACATCGTATGGGTATTCTC CCTCATTCAGATCCCACAG

*Underlining indicates restriction enzyme sites.

**Undulating lines indicate the coding sequences for FLAG tag or HA tag.
